# Beyond Indicators and Success Stories: An Emerging Method to Assess Social Learning in Large-Scale Transdisciplinary Research Programs

**DOI:** 10.3389/fsoc.2021.649946

**Published:** 2021-06-22

**Authors:** Ying-Syuan Huang, Blane Harvey

**Affiliations:** ^1^ Department of Integrated Studies in Education, McGill University, Montréal, QC, Canada; ^2^ United Nations University Institute for the Advanced Study of Sustainability, Tokyo, Japan

**Keywords:** social learning, climate adaptation, large-scale programs, participatory evaluation, generative causality, contribution analysis method

## Abstract

Facilitated learning approaches are increasingly being used as a means to enhance climate and sustainability collaborations working across disciplines, regions, and scales. With investments into promoting and supporting inter- and transdisciplinary learning in major programs on complex global challenges like climate change on the rise, scholars and practitioners are calling for a more grounded and empirical understanding of learning processes and their outcomes. Yet, methodologies for studying the interplay between learning and change in these initiatives remain scarce, owing to both the “hard to measure” nature of learning and the complexity of large-scale program implementation and evaluation. This paper proposes a new method for studying social learning in the context of large research programs. It aims to analyze the social learning of researchers and practitioners engaged in these programs and assess the contributions of this learning to the resilience of the natural and social systems that these programs seek to influence. We detail the theoretical basis for this new approach and set out six steps for developing multi-layered contribution pathways and contribution stories with stakeholders to document both the process and outcomes of social learning. The proposed method, we argue, can strengthen our analytical capacity to uncover the structural drivers and barriers to social learning that are often masked by the complexity of large-scale programs. An illustrative example, drawn from a large-scale climate adaptation research program, provides evidence on how this method might advance our methodological strategies for studying learning in these programs. We conclude by highlighting two key methodological contributions brought about through this approach, and by reflecting on opportunities for further methodological development. Enriching our understanding of learning and change processes, we argue, is an important avenue for understanding how we can pursue transformations for sustainability.

## Introduction

Global challenges such as climate change and sustainable development are characterized by their complexity. Addressing them requires collective coordination and action to deal with the interrelated social, environmental, and economic dimensions of the challenge and their associated nonlinear feedback systems. This recognition has led to a push for new modes of collaboration, coordination, and learning within international research programs, with a view to enhancing collaboration across disciplines, regions, and scales (Leemans, 2016; Cundill et al., 2019; Currie-Alder et al., 2020). Social learning (SL) is one such approach to addressing the need for enhanced collaboration and learning that has been taken up in numerous major research programs on climate change in recent years (Kristjanson et al., 2014; Harvey et al., 2017; Cundill et al., 2018).^1^

Social learning emerged as a field of study in natural resource management and environmental governance in the 1990s (Rodela, 2011). This form of learning, where *changes in understanding occurring at the level of individuals* lead to *changes in practice within wider networks and systems*, is also said to be a powerful tool in navigating complex challenges like climate change (Collins and Ison, 2009; Reed et al., 2010). Yet, the development of methods for assessing SL processes and their outcomes within these programs remains slow (Ensor and Harvey, 2015; van Epp and Garside, 2019). This is, in part, due to the complexity of both large multi-partner programs, and of learning and social change (Buffardi et al., 2019). However, failure to effectively assess SL and its contributions to wider programmatic outcomes can limit our ability and inclination to invest in the strengths of these approaches, and our understanding of what their limits might be.

To confront this challenge, we piloted an adapted model of contribution analysis*—*an evaluation method used to assess the contribution of an intervention to an observed outcome—to study the influence of SL on program processes and outcomes in large research programs focused on climate change and resilience. Our study focused on documenting and analyzing the learnings of researchers and practitioners who implemented the programs, though future analyses could also engage wider stakeholder groups. Our revised method of contribution analysis sought to document and distinguish between two fundamental and interrelated processes: program implementation, and social learning dynamics. This paper describes how we modified the method for these ends, reports on the results of a pilot case of application, and reflects on the potential significance of this approach. We first discuss the methodological challenges that are posed by the dynamic nature of SL and the complexity of the context within which its participants are situated. We then offer a detailed description of the method along with an illustrative example of application and reflect on the benefits and challenges presented by this approach. In doing so we seek to further expand the “methodological toolbox” (Tschakert and Dietrich, 2010) for understanding the interplay between learning and change in large-scale programs; dynamics that are often obscured and therefore difficult to study.

## Background and Literature Review

### The Rise of Learning in Large-Scale Transdisciplinary Research Programs

The growing interest in SL in large-scale programs, and in programs studying climate change and sustainability more specifically, arises from two concerns related to understanding and acting on complex challenges. First, there is a widespread recognition of the “wicked” nature of sustainability challenges and uncertainty concerning how to best respond to them (Turnpenny et al., 2009; Rodela and Swartling, 2019). “Learning to learn” collectively (Fazey et al., 2007) is seen as a key to managing this uncertainty by helping participants to enhance their adaptive capacity (Butler et al., 2015), build socio-ecological resilience (de Kraker, 2017), and support knowledge co-production (Chaffin et al., 2016). Secondly, SL is seen by many as an essential feature of participatory decision-making and collaborative governance (Pahl-Wostl, 2009; Berkes, 2017). Given the increasing emphasis on transdisciplinary and use-oriented approaches to climate and sustainability research, this evidence suggests that principles of SL may represent a key strategy for effective program implementation (Gerlak and Heikkila, 2011; van der Hel, 2016; Cundill et al., 2018).

With international investments into large-scale programs on climate change research on the rise—particularly in the Global South (Buchner et al., 2015), interest in the contribution of SL to the intended outcomes of these programs is growing. Research funders, among other stakeholders, are keen to understand whether investments into more learning-centered approaches to program design will result in tangible improvements in terms of outcomes, and if so, in what manner (Dexis Consulting Group, 2020). Researchers and facilitators of SL processes also recognize the persistent challenges related to structuring and facilitating SL across a range of contexts––particularly where power asymmetries are present (Muro and Jeffrey, 2008; Ensor and Harvey, 2015; Fisher and Dodman, 2019). Thus, there is broad consensus that “a more solid and empirically grounded understanding of social learning processes […] is needed to capture the essence of transformative change” (Suškevičs et al., 2018, p. 1101). Addressing this gap requires a better understanding of the relationship between learning and change in large programs, and how and why collective action can emerge through learning-oriented engagement (Newig et al., 2017; Paz-Ybarnegaray and Douthwaite, 2017).

However, widely adopted methods for opening up the “black box” of SL processes within large-scale programs remain scarce, particularly in the fields of climate change and sustainability. Ensor and Harvey (2015) review of SL practices in climate change adaptation noted an absence of methods for assessing SL outcomes at the systems scale, the very scale that many large programs seek to influence. Concerns about the lack of evidence about the factors that influence the learning process, or on how learning has contributed to the processes of transformative social change (e.g., policy and institutional changes, environmental effects) persist (Lebel et al., 2010; Gerlak et al., 2017; Cundill and Harvey, 2019). Many argue that the field continues to be “fuelled by recurrent, but often untested, assertions” about the benefits of SL (Cundill and Rodela, 2012, p. 7). This study, and the method it advances, seeks to address this gap.

### Challenges to Studying Social Learning in Large-Scale Programs

Effectively documenting SL processes to assess their influences on large-scale programs presents challenges on several fronts. Buffardi et al. (2019) point to four “hard to measure” dimensions of assessing change in large-scale programs, all of which feature, to varying extents, within evidence on programming for climate change and sustainable development:

(1) Multi-dimensional constructs, processes and outcomes (such as SL) are often intangible, making it difficult to identify representative indicators and appropriate instruments data collection;
(2) Challenging settings with many actors and fast-changing contexts yield external factors which are likely to significantly influence processes and outcomes;
(3) Multiple, uncertain pathways of change make causal inferences unreliable, as they introduce non-linear changes and confounding variables that are difficult to account for; and
(4) Diverse interests and aspirations characterize these partnerships, often with competing rationales and priorities for what should be studied and how.

These dimensions feature in recent studies of climate change and SL, with authors highlighting the non-linear nature of the processes (Kristjanson et al., 2014), the unpredictability of outcomes (Sol et al., 2013), and the challenge of establishing causal relations (Fisher et al., 2015; Fisher and Dodman, 2019). This emergent and unpredictable character sits at odds with time-bound programs, for which results must typically be observed and documented within a strict time frame. These recent studies also highlight the complexity and diversity of partnership configurations and implementation contexts within which learning must be studied (Harvey et al., 2017). For instance, the CARIAA program (discussed below) sought to promote learning and collaboration between roughly 450 researchers based in more than 40 organizations in 17 countries, over a period of nearly seven years (Cundill et al., 2019). In dynamic contexts like these, purely quantitative measurement, or retrospective assessments of SL against a predefined theory of change or key performance indicators are likely to be inadequate for understanding how and why learning and changes occur––key questions that could inform future program design and planning (Junge et al., 2020).

## Study Context and Rationale for Contribution Analysis

The research within which this method was developed and tested examined the role of SL processes in two climate change adaptation research programs implemented in Africa and Asia.^2^ This research aimed to understand whether, and how, researchers’ and practitioners’ learning within these programs contributed to network- or system-level changes, and ultimately to wider outcomes and impacts. The programs under study were two major climate change research initiatives: the Collaborative Adaptation Research Initiative in Africa and Asia (CARIAA; 2012–2018) and Future Climate For Africa (FCFA; 2015–2019). Both programs explicitly sought to embed SL processes into their work with a view to enhancing collaboration and impact, albeit in different ways.

The present study builds on earlier reflections conducted in both programs, which observed that facilitated SL was an important contributor to effective collaboration (Cundill et al., 2018; Jones et al., 2018; Koelle et al., 2019). We extend this analysis to focus upon one longstanding, but under-examined hypothesis around SL in transdisciplinary collaborations: the assumption that SL not only strengthens collaboration in large-scale programs, but also leads to discernable improvements in the programs’ outcomes and impacts. The method introduced in this paper seeks to advance the tools and evidence with which we can test that assumption, and ultimately help us to better ascertain how collective learning contributes to research and action on climate change.

For the development of this method, we selected five cases of change for analysis from the two programs using the steps set out below. Cases were identified and prioritized through a series of interviews (*n* = 4) and, in the case of the FCFA program, a survey of program members (*n* = 72). A further 15 semi-structured interviews were conducted for the contribution analyses of the selected cases. Participants were identified through snowball sampling based on the set of cases identified. The analyses were then validated with interviewees in line with the methodological steps we describe below. The methodological description that follows reflects the learning from those five cases. We have then selected one illustrative case to describe the process (*Illustrative Example: Social Learning Impacts in Botswana Through the CARIAA Program* below).

### Contribution Analysis for Social Learning Studies

The starting point for developing this methodological approach was with contribution analysis, an evaluative approach that has been increasingly used to examine causal issues in complex settings, particularly in the context of international development (Fisher et al., 2015; Koleros and Mayne, 2019). Contribution analysis offers a structured approach to evaluating the extent to which an intervention has contributed to an observed outcome (Mayne, 2012). The method was originally developed to address a key weakness of program evaluation; namely that outcomes or impacts are often reported with limited discussion as to whether (or why) they are the result of a specific intervention (Mayne, 2008; Mayne, 2015). Contribution analysis is thus aimed at producing “credible claims on the intervention as a contributory cause” of a program outcome (Mayne, 2019, p. 174).

Contribution analysis adopts the perspective of generative causality—seeing causality as a chain of cause-effect events (Pawson and Tilley, 1997; Gates and Dyson, 2017). This view holds that an intervention is composed of a series, or several series, of causal steps between program activities and their desired contributions (Mayne, 2019). The causal links of these steps form an impact or contribution pathway (or pathways) through which particular activities or interventions contribute to observed outcomes. More recently, scholars and evaluators using contribution analysis have sought to extend the approach to contexts such as large-scale initiatives that are characterized by greater complexity, more emergent program design, and with “whole systems” orientations (Junge et al., 2020). Here, contribution analysis can be used to produce “thick” descriptions (*sensu* Geertz, 1973) of change processes that unfold over extended periods of time, attending to multiple strands of change, and accommodating the interpretations of multiple actors engaged in the activities under study (Junge et al., 2020).

Examined through contribution analysis, SL can be understood as interventions in a large-scale program, encompassing multiple learning processes and intermediate outcomes which together form a contribution pathway. By focusing on the contribution pathways of SL, instead of assessing learning activities in isolation, we are able to draw distinctions and connections between effective learning processes, program implementation activities, and program outcomes––recognizing that the causal relations between these strands may be partial at best. We are also able to gain insights about the cumulative contributions of SL in large-scale programs. Importantly, the analysis process allows us to 1) reveal the learning dynamics in large program processes over time, 2) demonstrate the *causal links* between contribution pathways and program outcomes, 3) assess the extent to which SL is a *necessary* ^3^ *contributory cause* of the observed programmatic outcomes and impacts, and 4) build *causal narratives* of why and how SL has contributed to the wider program influence (Mayne, 2019).

**FIGURE 1:**
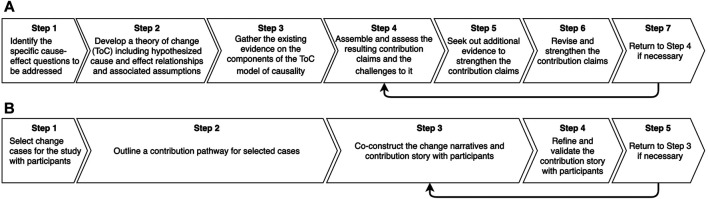
Steps of Mayne’s (2011) contribution analysis and the modified procedure used in this study. Figure 1 compares Mayne’s (2011) contribution analysis to the steps that were used to assess the contribution of social learning to programs’ outcomes and impacts in this study.

The original contribution analysis approach set out by Mayne (2011) involves seven steps (Figure 1A). Although this step-by-step procedure seems appealing, evaluation scholars and practitioners are still grappling with its actual implementation (Lemire et al., 2012; Dauphinee 2015; Riley et al., 2018). Mayne (2015) also calls for more rigorous and feasible methods for analyzing the non-linear contributions of a complex intervention to a change process. Recent attempts in this direction adapt contribution analysis by, for example, using nested “theories of change” to account for multiple contribution pathways (Douthwaite et al., 2017; Koleros and Mayne, 2019) or constructing sub-theories of change to capture the “emergent and unstable” interventions in large-scale, transformative change processes (Junge et al., 2020, p. 235).

The adapted contribution analysis we propose here, which is discussed in detail in the *An Emerging Method to Assess Learning Contributions* section, reconceptualizes the method’s original theory-based underpinnings by modifying the second and third steps of the original procedure (Figure 1B). More specifically, the framing of both SL and contribution analysis in this study adopts a constructivist perspective on knowledge creation. This epistemological perspective emphasizes the central role program stakeholders must play in constructing and interpreting findings if we wish to achieve a robust understanding of complex social realities (Kushner, 1996). This grounding, we argue, strengthens the method’s sensitivity to the underlying drivers and barriers to SL processes. Moreover, building on theories of systems change, this methodological approach seeks to be explicit in distinguishing between isolated change “events” (meetings, trainings, policies being adopted, etc.) and evolutions in deeper patterns, system structures and mental models that emerge from SL processes (see Senge, 1990; Sheffield et al., 2012).

### Roles and Participation in Social Learning Contribution Analysis

Large-scale programs like CARIAA and FCFA tend to span scales, geographies, and contexts––from community-scale work led by NGOs and extension agents, to international policy engagement led by intergovernmental agencies and research organizations based far from the sites where program activities are being implemented. This can make the identification of a representative sample of participants challenging. Given the method’s dual focus on 1) program implementation, and 2) the SL processes and outcomes that shape that implementation, participation is needed from actors who can speak to both processes. In many cases there may be considerable overlap in these roles, with program managers promoting SL processes as a means of supporting collective reflection and action, for instance. This reduced the overall number of participants needed to meet these various information needs.

The types of participants required may also vary according to the stage of the contribution analysis process. The early steps of case identification and contribution pathways call for participants with a good overall knowledge of program activities, the assumptions behind the program design, as well as overall program governance. Later steps of co-constructing detailed narratives of the cases under study, conversely, requires participants with detailed first-hand knowledge of how these processes unfolded, both formally and informally. Their perspectives on the programs’ outcomes and impacts are particularly important in documenting the reality of how program processes unfolded in practice, and for drawing connections between learning and wider development and sustainability outcomes. All participants contributed to the process of constructing the change narratives through interviews or group discussions.

## An Emerging Method to Assess Learning Contributions

This section provides a stepwise description of the methodological approach (Figure 1B) taken to assessing the contributions of SL to specific outcomes in the CARIAA and FCFA programs. Our rationale for modifying Mayne’s (2011) contribution analysis is also discussed. As we advance to describe the method, we clarify some of the terminology that will be used in Table 1.

**TABLE 1:** Definitions of key terms.

Term	Description
Change case	Changes that occurred in the program setting that influenced overall program outcomes or impacts. These are the cases identified from program documents and program participants that are subjected to analysis
Change narrative	Individuals’ descriptions of how the change case being studied unfolded. This is grounded in individual experience and memory
Contribution claim	A description of a causal link, where a particular action, event, or product contributed to a specific, intermediate outcome
Contribution pathway	A set of causal linkages in the sequence of steps that take program activities to impacts (Mayne 2019)
Contribution story	The end-product of a contribution analysis. Contributions stories provide rich and evidenced descriptions of the contribution pathways that led to a particular change. These are generated through analysis of multiple change narratives and supporting documentation of the change case under study

### Step 1: Selecting Change Cases for Study

The first step of Mayne’s (2011) contribution analysis is to set out the specific cause-effect questions to be addressed. We have divided this step into two parts aimed at identifying robust cases, and clarifying their definition in dialogue with program members. Given our aim of understanding how SL has contributed to program outcomes, we begin by identifying all candidate cases where this may have happened. The change cases are identified through a participatory process of elicitation, inspired by the “most significant change technique” (see Dart and Davies, 2003), as well as an analysis of program reports and related documents highlighting significant program outcomes. In line with the “most significant change” technique, the focus of this method is not to assess whether or not SL has occurred in the selected cases, but rather, how it has taken place and how it has contributed to the selected change cases.

Once a list of candidate cases has been developed these are scored using two criteria to ensure they are aligned with the objectives of the contribution analysis: 1) relevance of the change to program objectives, and 2) perceived contribution of SL to the change in question. Again, this scoring involves the participation of program members who score candidate cases according to these criteria, sometimes making suggestions on how the framing of the cases could be clarified, or where individual candidate cases should be combined to produce a larger, more coherent example of change. Early engagement with the intended audience (program managers, principal investigator, and facilitators of SL in our study) in problem framing and focusing can improve accessibility and perceived usefulness of the findings being communicated (Arevalo et al., 2020). Once the scoring is complete, participants select the cases that they think warranted further investigation.

### Step 2: Outlining a Contribution Pathway for Selected Change Cases

**FIGURE 2:**
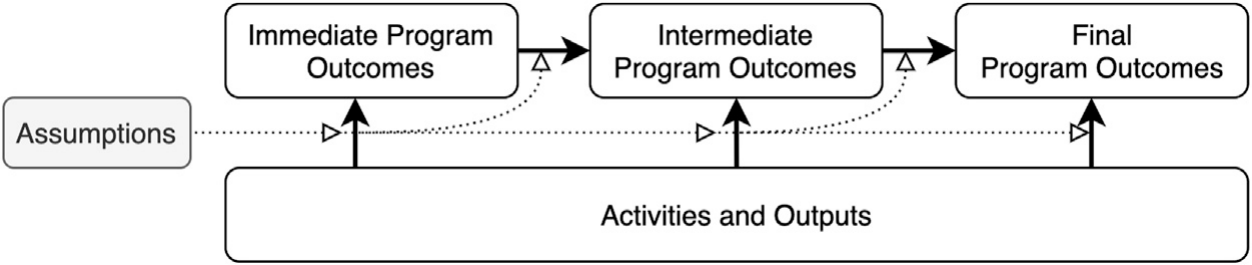
A generic contribution pathway. Figure 2 illustrates the conceptual model in Mayne’s theory of change.

The second step of Mayne’s (2011) contribution analysis method is to develop a theory of change which describes a causal pathway between specific activities and their outcomes or impact, as well as the assumptions that informed the steps in that pathway (see Figure 2). Mayne (2019) notes that theories of change tend to be based on established social science theories “so that they can provide the basis for solid causal explanations” (p. 183). However, a deductive approach to data collection can limit our ability to uncover the structural drivers and barriers as well as the informal and emergent practices of SL, such as trust-building, coalition-building, and the processes of negotiating meaning. Moreover, as discussed at the outset, a lack of empirical evidence on learning dynamics in large-scale programs further complicates this task. Capturing both program implementation activities as well as SL processes within a common theory of change is particularly challenging because the two processes do not necessarily unfold in direct parallel. Condensing these change dynamics into a single pathway also constrains our ability to illustrate the emergent and iterative influence of SL on program processes and outcomes.

**FIGURE 3:**
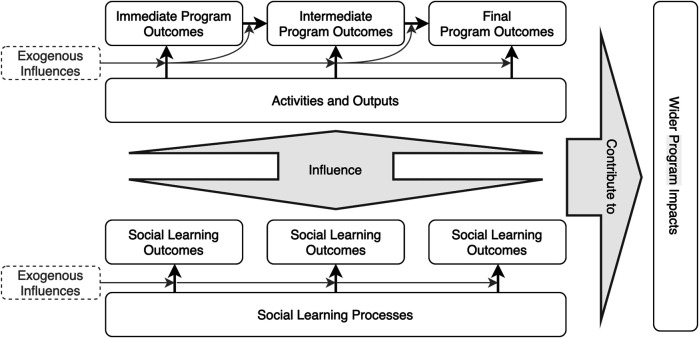
A two-layered contribution pathway capturing social learning dynamics. Figure 3 illustrates the conceptual model of the proposed method. It splits the contribution pathway into a program implementation layer and a learning-focused layer to outline the change process.

To address this challenge, we modified the step by developing contribution pathways that are divided into two layers (Figure 3). The first layer illustrates the program implementation process for the change case in question, describing the sequence of events, outputs, outcomes and impacts that constituted the case. The result is a tentative timeline of the change process and postulated causal connections between these elements of the pathway. The second layer focuses on the learning dimension of the change process, or what we sometimes called the “behind the scenes” narrative of the SL that took place and its impacts. It consists of three components, namely 1) learning goals (e.g., to ensure the continuity of a working group), 2) learning processes (e.g., regular check-in calls, jointly setting a meeting agenda, ongoing tailored trainings), and 3) learning outcomes (e.g., established a norm of mutual engagement, applied the learned knowledge and skills in the research process).

The objective of this second step is to generate a tentative description of the change process under study, which can guide the interviews and participant validation in the subsequent steps. The contribution pathways are developed through a combination of document analysis and preliminary interviews with participants to document their change narratives. In the pilot case described below, the documents studied included annual reports, partner reflections and stories (such as blogs), academic publications, evaluations, and learning reviews. While constructing a contribution pathway with two layers allows us to begin illustrating the dynamics between SL and program processes, it is worth noting that the learning-centered pathway tends to be absent or highly fragmented at this early stage because evidence on SL is rarely systematically documented throughout the program period. There may also be multiple explanations about the causal links between change events, program outputs and outcomes in the documents because they are recorded by different actors, from different standpoints, at different stages of the program process. These issues are addressed in the subsequent steps.

### Step 3: Co-Constructing Contribution Stories With Participants

Narrative evidence on SL processes and their outcomes are crucial for this constructivist approach to contribution analysis, especially when there are gaps in documentary evidence. However, perspectives on learning processes and their contributions to change are strongly tied to participants’ own vantage points within these processes, their personal assumptions, and the depth of their engagement in the processes. Therefore, the contribution pathways developed in the previous stage must be further co-developed and tested in order to strengthen the robustness and perceived credibility of their contribution claims. To this end, participants with in-depth knowledge of the change cases are invited to contribute their own change narratives and aid in co-constructing the contribution pathway and story. This participatory process responds to concerns that contribution analysis, as a theory-based evaluation method, tends to overly privilege the views of researchers and evaluators (Rogers, 2008). It also tends to result in a considerably more complex depiction of the change process (Junge et al., 2020).

In the five case studies used to pilot this method, the authors carried out 3-8 individual or small group interviews with key actors from each change case, either face-to-face or virtually. In these interviews, participants are invited to review the draft contribution pathway diagram that is crafted as an editable timeline of events and processes. Participants are also asked to share their own change narrative, identifying aspects of the pathway diagram that are incomplete, unclear or inconsistent with their recollection or interpretation of the events. Emphasis is placed on having participants describe the drivers and impacts of particular stages in the change process through the use of probing questions such as: “What led that to happen?” and “why do you think that moment was significant?” Importantly, participants are invited to challenge the authors’ causal inferences concerning the influence of SL on the change processes in order to enhance the credibility of the contribution claims set out in the pathway. The revised contribution pathway diagram and interview transcripts provide the authors with the basis for developing the contribution story.

### Step 4: Refining and Validating the Contribution Story

A key part of the researcher’s role in developing the contribution story is continual checking, questioning, and confirming the emerging details and contribution claims as they are identified. This strengthens the results, and thus the rigor, or trustworthiness (the term most often used in the naturalistic paradigm) of a study (Kvale, 1989; Morse et al., 2002). To this end, the authors adopt Lincoln and Guba, 1986; Lincoln and Guba, 1990) quality criteria in qualitative research, namely: credibility, dependability, confirmability, and transferability.

Credibility refers to the confidence of the truth of the contribution story. It is first enhanced through an ongoing and trusting engagement with the program members and study participants. Its effect can be observed in the first step of the method where only a small set of the change cases in each program are seen influenced by SL, and thus selected for further investigation. This process involves recognizing that some candidate cases did not lead to significant outcomes, or were not the product of SL interactions (based on the insights of participants with first-hand knowledge of how events unfolded).

The authors also seek to strengthen the credibility and dependability of the contribution story through an iterative process of assembling evidence and reassessing the contribution claims (Mayne, 2019). The focus of dependability is to ensure the consistency of findings over time. Here, critical reflection and researcher reflexivity are needed to assess the strengths and limitations of the contribution claims within each case. Researchers and participants can ask themselves: Are the contribution claims grounded in empirical evidence? Is the chain of results logical and in line with interviewees’ narratives? Whose perspectives may be missing?

Triangulation of claims across multiple sources of evidence can also be used to resolve any contradictions. In some cases, parts of a contribution story may differ across documents and interviews. When this occurs, the authors ensure that competing interpretations are kept, and interviewees are invited to comment on them. Through multiple rounds of data collection and comparison, we seek to establish the most plausible explanation supported by robust evidence and multiple actors’ interpretation of events. Importantly, final outputs of the research analysis (e.g., revised contribution pathway, contribution story) are shared back to the participants for validation and member-checking to ensure credibility and dependability of the study findings.

Confirmability is concerned with the degree to which the study findings could be confirmed by other researchers. This is addressed, in part, through process of member-checking described above. The authors also establish confirmability by transparently describing the research steps taken from the start of a research project to the development and reporting of the findings, as shown in the illustrative example below.

Finally, transferability is the degree to which the contribution claims can be transferred to other contexts or settings with other program members. While it is impossible to draw direct comparisons from one context-specific case experience to another in another setting—as one might do in more experimental designs—the use of existing scientific theories or frameworks can be used to test contribution claims within the story to further strengthen the confirmability and transferability of insights from the study. For example, Wenger’s (2000) communities of practice framework, commonly used in SL literature, offers an analytical lens to identify key causal links that have contributed to the overall pathways of SL and their influence on the emerging program outcomes in one of the FCFA cases.

## Illustrative Example: Impacts of Social Learning in the Collaborative Adaptation Research Initiative in Africa and Asia Program

To illustrate the use of this approach in practice, we present our analysis of a change case from the Collaborative Adaptation Research Initiative in Africa and Asia (CARIAA). The CARIAA program (2012–2018) was jointly funded by the United Kingdom’s Department for International Development (DFID) and Canada’s International Development Research Centre (IDRC) to support applied climate change research in 17 countries of Africa and Asia. CARIAA was composed of four research consortia, one of which was the Adaptation at Scale in Semi-Arid Regions (ASSAR) consortium, led by the University of Cape Town, which is the focus of this specific example. IDRC was the primary organization convening SL activities across the CARIAA program. Key members of IDRC who have professional interests in SL and transdisciplinary collaborations were thus invited to participate in the case study.

### Identifying and Selecting Change Cases

At the outset of the study, IDRC created a list of significant program impacts (or changes to which the program contributed), generated through a review of the four CARIAA consortia’s final progress reports. This list served as the start point for selecting cases of study for CARIAA.

The authors began the first step by developing a table for scoring options and asked participants to score the significance of each of the changes listed as described in the *An Emerging Method to Assess Learning Contributions* section discussed above. We reviewed the scoring table through one-on-one and group discussions with seven CARIAA members (e.g., program managers, principal investigators) who had either a broad overview understanding of the entire program and its activities, or deep knowledge of one of the four specific research consortia within CARIAA. We ensured that the set of participants included at least one person with detailed knowledge of each consortium. Through this process, three out of 28 cases were selected for development from the CARIAA program, one of which we will focus on below. This case, drawn from the work of the ASSAR consortium, is described as: *University of Botswana, Oxfam and University of Cape Town contributed to the District Development Plan for Botswana’s Central District*. Table 2 presents a brief background of this change case.

**TABLE 2:** Programmatic outcomes and impacts from the Botswana case.

A key area of emphasis in ASSAR was the uptake of research evidence into government planning and decision making. One way this uptake was encouraged was through a series of capacity building exercises, such as training in a participatory model of Vulnerability and Risk Assessment (VRA) designed to focus climate adaptation policies on the most vulnerable (Morchain et al., 2019). In Botswana, ASSAR’s VRA approach was particularly appreciated and was later adopted by the government for planning at district and national scales. It contributed to the capacity building of marginalized groups (e.g., unemployed youth) and research users (e.g., District Economic Planners, and the National Disaster Management Office based in the Office of the President). ASSAR team also contributed to drafting the District Development Plan for Botswana’s Central District (Bobirwa sub district) by adding a chapter on climate change.

### Contribution Pathways, Change Narratives and Contribution Claims

**FIGURE 4:**
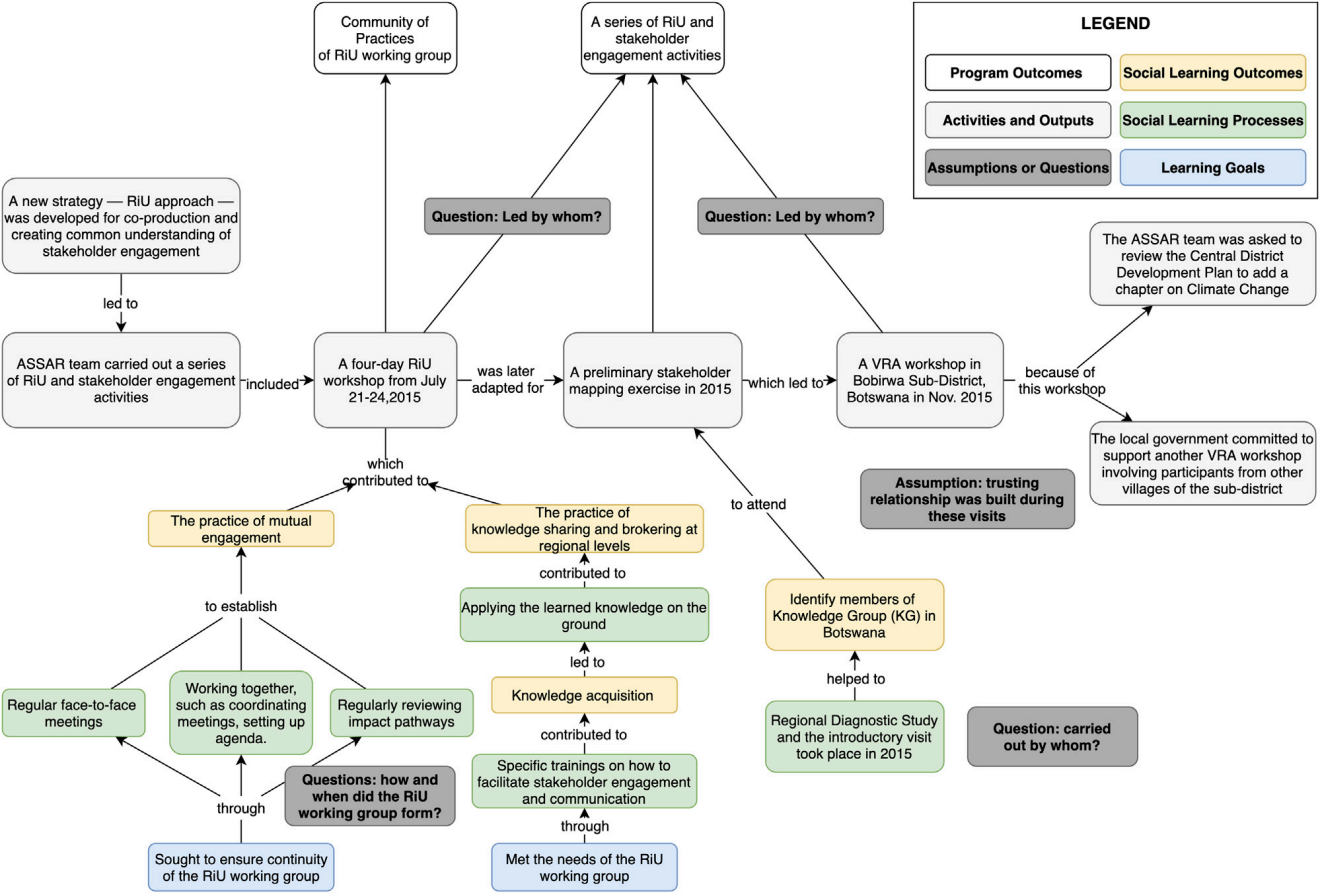
The initial contribution pathway based on document analysis. Figure 4 illustrates an intermediate step of our method.

Having identified the case, we then used a combination of document search and interviews to narrow down a set of documents as a starting point for developing the initial contribution pathway. In total, 73 documents were collected and screened, yielding eleven documents with content related to the case were retained. These served as the primary data for constructing the initial contribution pathway, presented in Figure 4.

Once a preliminary outline of the case had been developed through document analysis, we gathered change narratives through interviews with five participants who were integral to the case. The participants were identified through our document analysis and conversation with the CARIAA members. Each of them had different roles in ASSAR (e.g., facilitators, researchers, practitioners) and a different institutional affiliation, so we were particularly interested in their perspectives on how the collaboration and SL processes took shape, and how their sustained partnership led to the ultimate policy impacts described above. This use of multiple data sources allowed for triangulation, and thus strengthened the credibility of the contribution claims.

**FIGURE 5:**
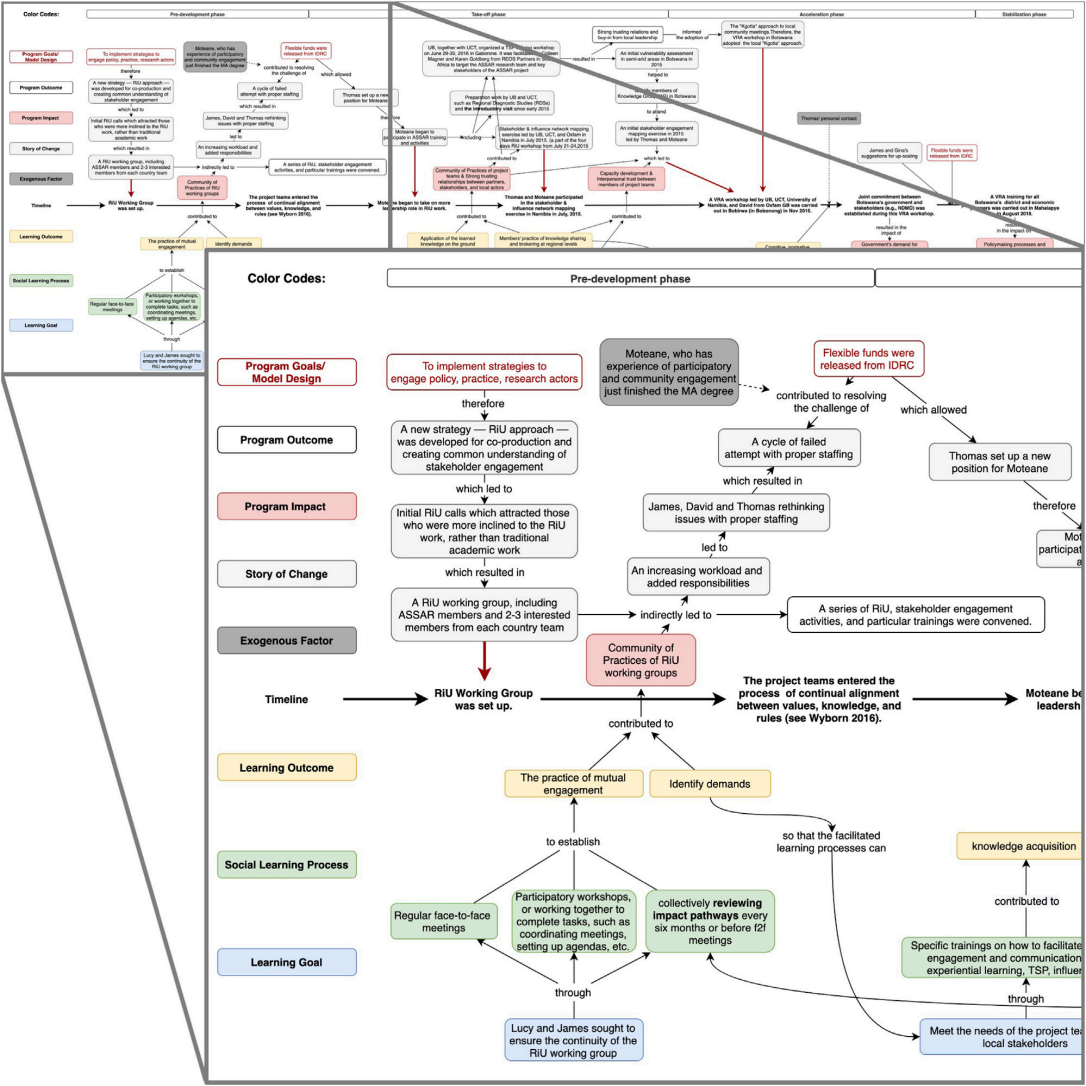
Excerpt of the finalized contribution pathway. Figure 5 is a snapshot of the finalized contribution pathway. The full contribution pathway of this change case is available at https://doi.org/10.6084/m9.figshare.14550840.v9. Key actors in this contribution pathway are two Oxfam-ASSAR partners (James and David), the Principal Investigator of ASSAR’s UB team (Thomas), and an early career researcher who later became the coordinator of ASSAR (Moteane). These are pseudonyms. The upper layer of the timeline illustrates the program implementation process for the change case in question, describing the sequence of events, outputs, outcomes and impacts that constituted the case. The bottom layer of the timeline illustrates the learning dimensions of the change process.

During the interviews, participants were invited to comment on the draft contribution pathway diagram (shared using GoogleDocs and the draw.io diagramming plugin). Attention was also given to understanding participants’ perceptions of the role of SL in the program implementation process. The authors then revised the contribution pathway diagram based on the interview transcripts (see Figure 5, full diagram available at Huang and Harvey, 2021).^4^ As Morse et al. (2002) note, “collecting and analyzing data concurrently forms a mutual interaction between what is known and what one needs to know (p. 18),” and this iterative process is the essence of attaining credibility of the study.

**FIGURE 6:**
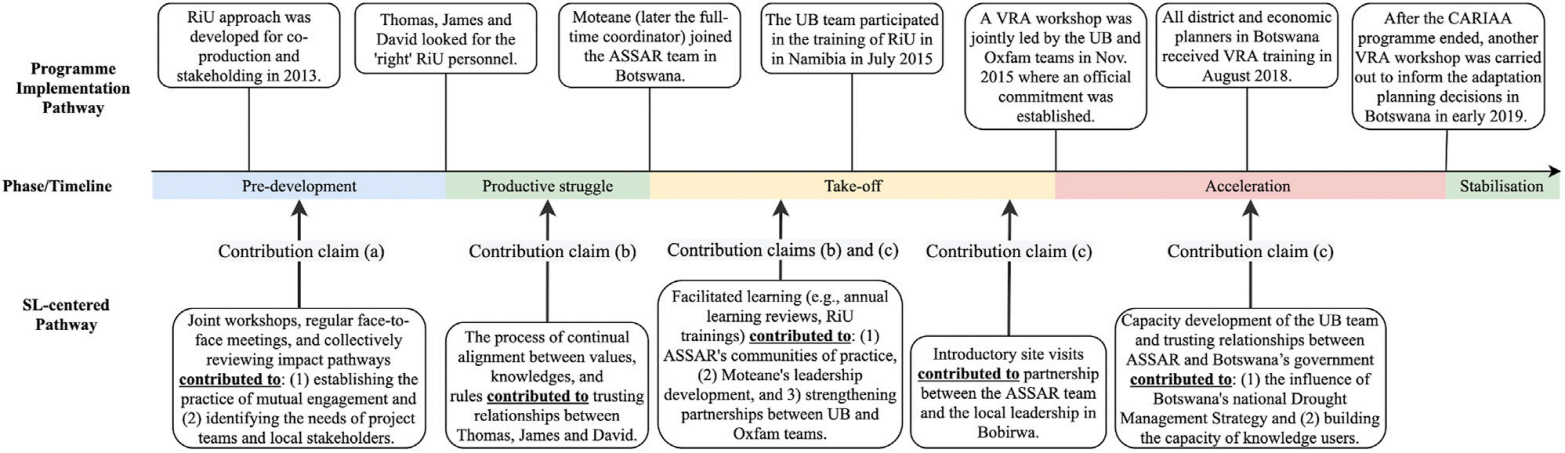
A simplified diagram of the contribution claims and change process in the ASSAR Botswana case. The contribution claims indicated in this figure are (a) tailored workshops contributed to mutual engagement, (b) prolonged engagement contributed to developing trust across partner organizations, and (c) ongoing learning contributed to new modes of pursuing research at UB and drought management in the region. The evidence on each contribution claim is presented in Table 3; Supplementary Material S2.

As a relevant output of the analysis, the finalized contribution pathway illustrates the added level of detail and complexity that emerge from the co-production process. The additional details provided much greater insight on the contribution of SL on program processes, as can be seen in the lower half of Figure 5. In order to highlight these learning dynamics from amidst the complexity particularly for sharing with wider groups of stakeholders, we simplified this updated contribution pathway into a contribution claims diagram (see Figure 6; Table 3) illustrating the points where SL contributed most directly to the program outcomes and impacts. To validate the contribution claims, as discussed in the *An Emerging Method to Assess Learning Contributions* section, all case materials were sent back to the participants for member-checking, which allowed for enhancing credibility and dependability of the study findings.

**TABLE 3:** Social learning contributions to the ASSAR Botswana case.

Title of the change case: Social learning processes contributed to the sustained engagement of high-level government personnel and ongoing collaboration between the Botswana Government, the University of Botswana (UB) and Oxfam
Key actors (pseudonyms): James and David (Oxfam-ASSAR partners); Thomas (principal investigator of ASSAR’s UB team); Moteane (early career researcher and later coordinator of the ASSAR’s UB team)
Contribution claims and associated scenes of the contribution story:
(1) Tailored workshops contributed to mutual engagement: The VRA methodology was adapted by the Oxfam team to strengthen Southern partners’ capacity to engage in user-oriented research-later called RiU, or research-into-use, Cundill (2018). James and David had initiated a RiU working group, but it “didn't quite gel” with many academic partners. After attending the first RiU workshop and “spending time” with James and David, Thomas “warmed up” to the concept. On a shared ride to the airport, David proposed an in-person visit to continue the discussion of finding a boundary organization to host the RiU person in Botswana.
(2) Prolonged engagement contributed to developing trust across partner organizations: After months of struggling without proper staffing, Thomas, with the support of James and David, negotiated for the creation of a special position and associated funding from IDRC to give Moteane additional responsibilities to play the brokering role as the RiU focal person. This process contributed to establishing interpersonal trust between the UB and Oxfam teams, as Thomas witnessed that James and David were “working along with [them] at every point” and “would come to agreement at every corner” to meet the needs of the partners and local stakeholders.
(3) Ongoing learning contributed to new modes of pursuing research at UB and drought management in the region: Thomas’ and Moteane’s in-person introductory visits contributed to the relationship building between the ASSAR team and the sub-district officers in Bobonong. These community leaders became the primary members of the knowledge group to co-plan and later participate in the VRA workshop in Bobonong in 2015. Jointly led by the UB and Oxfam teams, the workshop was received positively, resulting in an official commitment to adopting the VRA methodology into district level planning between the Botswana government and local stakeholders.

### Studying Social Learning’s Contributions Through the Change Case

Fisher and Dodman (2019) argue that, although examples of SL outcomes at individual, network- and system-scales can be found in cases they reviewed, “there is little discussion of how these scales link and how change happens across them or how the framing of scale preconfigures the type of changes sought and how that change might happen” (p. 245). The method we propose here begins to address these questions by illustrating the pathways of influence that SL has on program processes, from inter-subjective dynamics to wider systemic changes. Through this process, cognitive, normative and relational SL outcomes (Baird et al., 2014) were evidenced. For example, Oxfam and UB team members developed new collaborative capacities (Freeth and Caniglia, 2019) to engage with local stakeholders and government leadership and have maintained an ongoing partnership even after the end of the CARIAA program, representing both cognitive and relational learning outcomes. Moreover, the senior researchers in Botswana shared that the learning process has shifted their ways of doing research, toward an impact-focused, use-oriented approach to knowledge production in a lasting way (normative learning outcomes). This outcome is evident from their ongoing partnership and active engagement with the local government for the region’s drought management and adaptation planning after the CARIAA program.

Looking at the dynamics between SL and program processes in the case, the method serves to generate evidence supporting the assertion that careful program design and its associated learning spaces are key factors in creating the enabling conditions for SL (Cundill et al., 2019). For example, strategic partnerships between research bodies and NGOs (University of Botswana and Oxfam in this case), have been “integral to bringing policy, practice and impact considerations into all aspects of ASSAR’s work, which in turn enhanced both the diversity and impact of the consortium” (ASSAR, 2019, p. 30). Considering the dispersed nature of the consortium partnership, a careful design of face-to-face annual meetings and strategic virtual interactions between the ASSAR members contributed to the prolonged engagement between Oxfam and University of Botswana teams, allowing for partners’ continual alignment of needs, knowledge, and values for engagement through the project (Wyborn et al., 2016). Additionally, the flexible funds released by IDRC led to the creation of a new position for an ASSAR early career researcher to take on a leadership role in Botswana when the team was struggling to continue the work. This systemic factor was not only a turning point for the program implementation process, but also contributed to the SL process by strengthening the trust between ASSAR members. While this outcome may be dependent on the specific context where IDRC’s funds were applied, the cumulative effect of SL was an integral component of how the program members from diverse backgrounds could build on the existing trust and optimize the funding opportunity to expand the impact of their collaboration on policy change. These findings offer us an entry point to gain insights into the interplay between SL and change in large-scale program contexts.

## Conclusions and Further Considerations

One important trend in contemporary research on global challenges like climate change is a growing focus on the real-world impacts in the near term (Schuetz et al., 2017). Transdisciplinary learning within large-scale climate and development programs is seen as an important element of navigating the complexity of these challenges, linking research to action, and transforming systems and practices (Cundill et al., 2018). This is reflected in Eriksen et al. (2015) assertion that “opportunities for critical reflection and processes of social learning may hold the greatest promise for achieving the promise of transformational adaptation [to climate change]” (2015, p 530). However, this rising focus on embedding learning and collaboration within research programs has prompted calls for substantial improvements with respect to methods for studying learning (Rodela, 2012; Gerlak et al., 2017) and understanding its impacts (Fisher et al., 2015). This article responds to these concerns by proposing a means of better understanding the contributions of social learning (SL) to program activities.

The novelty of this emerging method is twofold. First, its constructivist orientation to contribution analysis disrupts the primacy of the evaluator’s role in interpreting the evidence available in favor of a more participatory mode of knowledge creation (Rogers, 2008). The boundaries between “researcher” and “informant” are blurred and a range of program members are involved in narrating and interpreting the cases of change under study, thus attending to the fact that perspectives on both learning and change are fundamentally influenced by one’s role and position in the process. The participatory approach set out above may enable a more direct uptake of findings among the practitioners and program managers (Arevalo et al., 2020) who will continue to lead the implementation of large-scale climate change adaptation programs in Africa and Asia in our case. As shown in our illustrative example, one main factor that contributed to the SL among the program members is the program’s careful design. As a result, the learning of individual researchers at University of Botswana has since been transferred into their networks where district planners adopted new modes of pursuing drought management in the region.

Secondly, the inclusion of multi-layered contribution pathways helps to distinguish and illustrate the dynamics between overlapping processes of SL and program implementation. This methodological strategy provides a much more nuanced picture of SL in complex settings where problems are “wicked” and transdisciplinary collaborations are needed. It also provides a way to generate robust evidence on the effects of SL within networks and systems, the scales that sustainability research and practice often seek to influence. As illustrated through our case study, analysis across the two layers of contribution pathways can provide a much clearer understanding of how a particular learning process shapes the dynamics of program implementation. This clarity can help program managers, researchers, and evaluators to distill evidence-informed lessons that are transferable for future program design. The modification of contribution analysis also helps us better detect the underlying factors, such as structural barriers and existing norms, that foster or inhibit the processes and outcomes of SL. It can therefore be invaluable when assessing the drivers of success or failure in large-scale programs. Finally, given the perceived link between learning and transformation, this method may also provide insights on how we can better embed the processes, structures, and ways of learning into programs in ways that allow us to move away from incremental forms of change.

With this said, no approach is without limitations. Reflecting on our experience, we conclude with a few considerations to stimulate further improvement and discussion on this emerging method:

(1) Collecting real-time program data: In their synthesis review, Ensor and Harvey (2015) found that most SL studies are case-based and rely on *ex-post* analysis of the learning outcomes rather than active documentation as the process unfolded. This practice often leaves out the vital reflexive component of qualitative analysis. As adult education theorist Griff Foley (1999) noted, “the process of critical learning involves people in theorizing their experience: they stand back from it and reorder it, using concepts like power, conflict, structure, values and choice” (p. 64). Without engaging reflexively in the research process, assumptions about the outcomes and alleged benefits of SL can remain unchallenged.
(2) Considering the time and costs required*:* Based on our experience, searching and synthesizing relevant documentations was especially time consuming because information about the processes and outcomes of SL in large-scale programs were not systematically documented throughout the program period. This issue can be a particular challenge when dealing with the interventions that hold broad working definitions or ambiguous conceptual boundaries, such as SL.
(3) Multimedia data collection, analysis and communication of findings: Given the growing demands for knowledge mobilization, there exists a large amount of multimedia information (e.g., digital storytelling, Twitter feeds) produced and shared by program stakeholders and partners. Integrating tools and methods to analyze these sources could represent an opportunity to gain added insights into the members’ SL experiences, or to compare and aggregate insights in novel ways. New technologies can also be used for developing and revising the contribution pathways and contribution stories with participants in real-time, allowing for more open dialogues and negotiation in a knowledge co-production process. The approaches described here begin to use these kinds of collaborative technologies, but there is further room to innovate. To maximize their reach and influence, exploring ways to use multiple formats to share the contribution stories is also needed.

## Data Availability

The original contributions presented in the study are included in the article/Supplementary Material, further inquiries can be directed to the corresponding author.

## Footnotes

1 Examples of recent large-scale climate and development programs that have sought to integrate social learning principles and processes into their design and implementation include: The CGIAR’s Climate Change Agriculture and Food Security program (CCAFS); the Climate and Development Knowledge Network (CDKN); the Building Resilience and Adaptation to Climate Extremes and Disasters program (BRACED); the Collaborative Adaptation Research Initiative in Africa and Asia (CARIAA); and Future Climate for Africa program (FCFA).

2 Ethics approval for this research was granted by McGill University’s Research Ethics Board (ethics certificate #347-0119).

3 In his recent work, Mayne (2015; 2019) began to integrate the concept of “likelihood” to assess the degree to which an intervention is a “likely necessary” factor of an overall impact pathway. This allows a probabilistic interpretation of a contribution cause in the overall change processes.

4 The figure can be downloaded at https://doi.org/10.6084/m9.figshare.14550840.v9.
