# Supplementary material for: Beyond Indicators and Success Stories: An Emerging Method to Assess Social Learning in Large-Scale Transdisciplinary Research Programs
Source: Front Sociol. 2021 Jun 22;6:649946. doi: 10.3389/fsoc.2021.649946 (PMC8258259; doi:10.3389/fsoc.2021.649946)
Supplement: Supplementary file 1 [file Table1.DOCX]

**S2. An example of outlining the evidence on each contribution claim**

| **Contribution Claim** | **Evidence** |
| --- | --- |
| Joint workshops, regular face-to-face meetings, and collectively reviewing impact pathways **contributed to**: (1) establish the practice of mutual engagement and (2) identify the needs of project teams and local stakeholders. | A survey that was run among ASSAR members in late 2016 indicated that one of the key highlights with regard to their experience of working within ASSAR was tied to their new way of thinking about impact, communications and the societal benefits of their research. This was achieved through team workshops on R4I approaches and influencing, webinars, and exposure to tools like VRA, TSP, stakeholder mapping and power analysis. (Prakash et al., 2019, p. 19-20).    The impact pathways started as the tool, it's actually the tool itself that generates the conversation [...] it was kind of typical fashion where we drove the overall idea and asked people what they really wanted out of it. So we said, 'what we want now is we want to help you with planning the influencing phase of our work, which is kind of now that the research is coming out how we're actually going to use it and now that we've been positioning ourselves for the last years, what are we going to do with it. (Interview) |
| The process of continual alignment between values, knowledges, and rules **contributed to** trusting relationships between partners. | After the first few interactions, I really felt like in order to have a voice in this project, we are going to have to do something really different, or we're going to have to push boundaries or try to somehow make it known that the we want to contribute in a way that is beyond the initial idea. (interview)    Trust is very critical. Honesty I suppose. Trust and honesty, probably go together. [...] When that fell through, we went to a second organization, which is a much bigger organization. [...] But its independence is questionable. [...] and in three months, she resigned. [...] So once again, that fell through. And they gave us one of their staff members [but his] delivery was very close to zero [...] in the end we went back to the default, which was, UB team through Moteane to champion the RiU work. [...] And to Oxfam credit, David and James were quite working along with us at every point and were engaging to a point where we'd come to agreement at every corner. (Interview) |
| Facilitated learning (e.g., annual learning reviews, RiU trainings) **contributed to**: (1) establish a Community of Practice, (2) develop leadership, and 3) strength trusting relationships between partners and project teams. | See Molefe (2016)    One thing that was key to success were the face to face meetings, whether they be the RiU workshops or the annual meetings, and the reason it was so fundamental to success was the idea of building trust and relationships. (Interview)    The RiU working group [...] was kind of the [...] backbone for this project. [...] something that's lasted for the five years and they were meeting weekly or biweekly calls, involving all of the different regions, So there was a very kind of a dedicated the accompaniment of these processes and recognizing also that each region was so different and the opportunities in each region were incredibly different. (interview)    James came over to Botswana. And went out to the field. We talked about the VRA. And James gave the idea that "no, no, no, no, if you are going upscale, you only have the level of subdistrict. Maybe let's go to a full district level." So they are James's idea and that was in December of 2015. And were planning this and planning that, different activities for ASSAR until we had an annual meeting in Ethiopia in 2016. And it was [after] that meeting when Gina visited the project area in Botswana that Gina said, "why don't you upscale this to a higher level if you can do it, and gain training of planning officers at a higher level." That's all she gave us as a challenge. And we said, "well, it was worth thinking over." So Moteane and I, our team at UB. Why don't you try this at the national level? So that's when the international training concept coming in. Davie, Moteane and I worked on this until last August 2018. (Interview) |
| Introductory site visits **contributed to** partnership between the ASSAR team and the local learnership in Bobirwa. | Moteane and Sara went into the field in Bobonong, in the local districts to now talk to identify who are the key players, either key players in terms of making decisions or key players in terms of impact of decisions. [...] And it is from that visit, which was to me possible because already we had built up trust with the local admin staff in the traditional leadership. [...] the local people now with an idea of, oh, you are the guys who came earlier, now you've come to implement, we are working with you. (Interview)    In Botswana it is customary to discuss issues of development during “Kgotla” (community) meetings. [...] The VRA adopted this approach in that it brought together a diverse range of stakeholders (in institutional, sectoral, social, economic, educational capacities, as well as gender, age and interest) to discuss their ideas and experiences openly and freely. [...] Based on this, the local government leadership – Assistant District Administrator and the Senior Assistant Council Secretary – hailed the VRA workshop and its output as veritable and readily usable for sub-district planning. (Molefe and Masundire, 2016, p. 6) |
| Capacity development of the UB team and trusting relationships between ASSAR and Botswana’s government **contributed to**: (1) influence Botswana's national Drought Management Strategy and (2) build capacity of research users. | One of the strengths of the VRA workshop in Botswana was how it enabled people to see the links between their activities and climate risk and responses. […] When the Assistant Council Secretary for Bobirwa Sub-District closed the meeting she said: ‘I believe you will all go out from here as change agents and share with those who weren’t able to spend time here.’ (Masundire et al., 2016, p. 42)    See also Morchain et al (2019) and Rao (2018). |
